# The barriers and facilitators of Iranian men's involvement in perinatal care: a qualitative study

**DOI:** 10.1186/s12978-022-01350-9

**Published:** 2022-02-21

**Authors:** Sepideh Hajian, Nahid Mehran, Masoumeh Simbar, Hamid Alavi Majd

**Affiliations:** 1 Midwifery and Reproductive Health Research Center, School of Nursing and Midwifery, Shahid Beheshti University of Medical Sciences, Tehran, Iran; 2 School of Nursing and Midwifery, Qom University of Medical Sciences, Moallem Street, Qom, Iran; 3 School of Allied Medical Sciences, Shahid Beheshti University of Medical Sciences, Tehran, Iran

**Keywords:** Men, Involvement, Perinatal Care, Qualitative Research

## Abstract

**Introduction:**

Pregnancy and childbirth are crucial events in women's lives that can be done well with the support of people around them, especially their husbands. However, a number of factors can reduce or increase the supportive role of spouses during this period. The aim of the present study was to explore the barriers and facilitators of Iranian men's involvement in perinatal care.

**Materials and methods:**

This was a qualitative phenomenological study that sampling of respondents (pregnant women or the women who have recently given birth (one week to six months after childbirth), spouses, policy makers and midwifery service providers) was done through purposive sampling. The inclusion criteria included: being Iranian, the ability to understand and transfer the concepts into Persian, and employment in a midwifery center for at least one year (for service providers). Data were collected through in-depth interviews until the data saturation. The collected data were analyzed by conventional content analysis based on Graneheim and Lundman method steps. MAXQDA version 10 software was used to manage the data and Guba and Lincoln criteria were also used to ensure the trustworthiness of findings.

**Results:**

Men's involvement in perinatal care was found to be influenced by certain incentives, particular constraints and some gender authoritarian attitudes. There were 5, 4, and 3 subcategories in incentives, constraints, and gender attitudes respectively.

**Conclusion:**

The results revealed that men face a dual mechanism in participating in perinatal care, in which some of these factors can facilitate their participation and others can reduce it.

## Plain language summary

Men's participation in the perinatal period can reduce the problems of this period and leave a good memory for the whole family. In this phenomenological qualitative study, through in-depth interviews, participants were asked to express their experiences and views on men's participation in the perinatal period and the factors that increase and decrease their participation. Finally, the data were analyzed using the proposed method of Graneheim and Lundman and MAXQDA software. Interviews were conducted with 21 people (data saturation limit) and the data were classified into 3 main categories and 12 sub-categories. The results revealed that men face a dual mechanism in participating in perinatal care, in which some of these factors can facilitate their participation and others can reduce it.

## Introduction

Pregnancy and childbirth is one of the most important and stressful periods of women's lives, which is associated with many physical and mental changes [1], but their ability to tolerate these changes and adapt to the stresses and hardships of this period increases with the support of others, especially the spouse [2]. The issue of men's involvement in the area of women reproductive health was first considered in 1994 at Conference on Population and Development (ICPD) in Cairo and later in the achievement of the Millennium Development Goals (MDGS) [3]. Despite the known benefits of men involvement in perinatal cares [4, 5] and direct and indirect its negative impacts, few men involve in maternal health services [6–8]. Studies are shown that factors such as absolute authority in decision-making, having power in financial resources, lack of required infrastructure, low awareness of maternal and neonatal health, cultural barriers, having a feminine attitude to perinatal cares, fear of negative judgments of others, inadequate work hours of providing health services, inappropriate attitudes of health staffs and the provision of non-friendly services in these centers are major barriers to men's involvement in perinatal period [9–12]. In Iran, some barriers including lack of awareness of women's care needs, embarrassment, belief in non-interference in women's affairs, femininity of health centers and lack of acceptance of men's presence in these centers, social stigmas and women's embarrassment of men's involvement in pregnancy and childbirth care has been confirmed [7]. In Iran, to achieve millennium development and sustainable development goals, men's involvement in affairs related to reproductive health and women's health has been considered in recent years, but unfortunately these programs in many health centers of Iran are not implemented in practice or their implementation and effectiveness are not monitored. As a limited number of studies have been conducted in Qom city of Iran so far on the men’s involvement in perinatal care [13] and no qualitative study has been done with the method of conventional content analysis in the field of barriers and facilitators of men's involvement, this study was conducted to explain the barriers and facilitators of Iranian men's involvement in perinatal care with conventional content analysis. The phenomenological descriptive studies describe the phenomena as they are experienced and seeks to clarify the essence of the phenomena experienced and to accurately describe them through the analysis of participants' experiences [14]. Content analysis method is also a systematic and objective way of describing phenomena aimed at identifying the goals, values, culture, and desires of the interviewees [15]. Thus, this study seeks to use this method to identify the barriers and facilitators of Iranian men's involvement in perinatal care so that appropriate interventions can provide to increase men’s involvement in these areas.

## Methodology

This was a qualitative phenomenological study that the participants who lived in Qom city, were pregnant women or those who had given birth recently (one week to six months after childbirth), married men and key informants (including midwifery service providers, midwifery care managers and policy makers). Qom city is located 170 km to the south Tehran, Iran, with an area of 285 km^2^ and a population of 1,200,000 people. In Qom, due to religious conditions, there are different ethnicities from different parts of Iran and a few countries around the world. They were selected by purposeful sampling method. The key informants were from public centers and the pregnant women and newly mothers and spouses were accessed through perinatal clinic or postpartum ward of Izadi hospital (one of the public hospital of Qom city).The inclusion criteria of the research included willingness to participate in the study, being Iranian, the ability to understand and express experiences in Persian and having at least one year of experience in units related to midwifery (for service providers). After explaining the present study, the researcher used in-depth semi-structured interviews and accordingly obtained information on the participants' experiences in the factors affecting men's involvement in prenatal and postpartum cares to identify barriers and facilitators of their understanding and experience. All of the subjects participated in the interview. Interviews were performed in March-August 2018 by the first author of this article (N.M), who was faculty member and a PhD student in reproductive health and had experience in conducting qualitative studies and is teaching midwifery students in health care centers. All stages of data recording and analysis were performed under the supervision of the corresponding author of this article (S.H.) as a faculty member with many years of experience in training and conducting qualitative research. The in-depth interviews were conducted in public hospital, public health centers, or any place where participants felt more comfortable. It lasted 30–90 min (average: 55 min) and was recorded by digital audio recording with the permission of the interviewees. During the interviews, Observation notes were written down in a note book. Before the study, a pilot was conducted that was not analyzed, but this is done to refine the guides.

Interview with women began with an initial question such as "Do you have any experience of your husband's presence with you during prenatal, childbirth, and postnatal period?" and continued with other questions, such as "How effective can men's involvement in prenatal, childbirth, and postnatal period be?". The similar questions were asked from men. Different questions were asked from key informants, based on the goals of the study. For example, "Do you have any experience of men’s presence with their wives during prenatal, childbirth, and postnatal period?". The interview process was clarified with exploratory questions such as "Can you explain more?". Immediately after each interview, the audio was heard several times and transcribed by the researcher. Also, if the participants were not satisfied with the recording, their speeches would be written on paper. Also, after coding each interview, the guide questions for subsequent interviews were changed, if needed.

The interviews continued until the information was saturated. After 15 interviews, no new data that led to the creation of a new class, were collected. However, 6 additional interviews were performed for further assurance. All of the participants continued the study, and none of the interviews required repetition. If needed, the interviews were returned to the participants so that they could provide feedback and possible changes. After each interview, qualitative data were analyzed based on the conventional content analysis method [17] and based on the steps proposed by Graneheim and Lundman [16] in which at the end of each interview, all the notes and the audio file of the interviews were typed word by word in the language of the interview (Persian). Then, the typed content was read several times to obtain a general understanding of their content. Semantic units and basic codes were determined. Similar initial codes were placed within the more comprehensive categories and the main themes of the categories (which included the main themes of the research) were determined [17]. In this section, MAXQDA version 10 software was used to manage the data.

Five Guba and Lincoln criteria were considered to ensure trustworthiness of qualitative results [16]. To increase the credibility of data, the methods of reviewing codes by other members of the research team, credibility of the researcher and his scientific and professional background, reflexivity of researcher, and searching for disconfirming evidence and prolonged engagement were used. To increase the reliability or dependability of the data, the interviews were carefully recorded and transcribed word by word. During writing the report, the participants’ conversations were cited and the study was reviewed by supervisors and experts. The code-recode method was also used to increase the reliability of the data. In order to increase the transferability of the data, rich descriptions method was used and the researchers tried to conduct interviews, especially the initial interviews, among the appropriate samples (those who had the highest experience and knowledge on the perceived needs). Also, the steps of the study were described accurately and clearly by stating the performed activities, so that other researchers could do it in other conditions and places. Confirmability of data was also achieved by reviewing reports and writings by at least two experts in the field of qualitative research and reproductive health and determining the degree of similarity in results of the studies.

This study, which is part of a PhD dissertation, was approved at Ethics Committee of Shahid Behashti University of Medical Sciences (code of IR.SBMU.PHNM.1394.284). ethical considerations of the study included obtaining the necessary permissions from the relevant authorities, obtaining the informed consent of the participants to participate in the study, assuring the participants about the confidentiality of their speeches, freedom of participants to leave the study at any time, determining the place and time of the interview based on the willingness of the participants, and not imposing any costs on the research subjects.

## Results

In the present study, 21 interviews were performed. A total of 5 pregnant or recently- delivered, 7 men and 9 key informants with an age range of 24 to 60 years and a mean age of 38.3 years (Tables 1 and 2) participated. After interviewing with the participants, 1856 initial code was extracted, which 33 final codes were obtained in the process of data analysis and comparison after classifying the codes and deleting similar codes. The main theme of the study was the "dual mechanisms of men’s involvement in perinatal care", which were classified into three main categories (including incentives, constraints, and authoritarian gender attitudes) and 12 sub-categories (Table 3). There were 5, 4, and 3 subcategories in incentives, constraints, and gender attitudes respectively.

**Table 1:** Demographic characteristics of pregnant women and women who have given birth recently and men who participated in the study

Variable	Pregnant women and women who have given birth recently	Men	Total
	N (%)	N (%)	N (%)
Age			
Under 40 years old	4 (80)	4 (1.57)	8 (6.66)
40 years old and older	1 (20)	3 (9.42)	4 (4.33)
Job status			
Housewives	1 (20)	0 (0)	1 (4.8)
Employee	4 (80)	4 (1.57)	8 (6.66)
Self-employed	0 (0)	3 (9.42)	3 (25)
Level of education			
Diploma and lower	2 (40)	1 (2.14)	3 (25)
Bachelor	2 (40)	5 (6.71)	7 (4.58)
Master and higher	1 (20)	1 (2.14)	2 (6.16)
Number of children			
0	3 (60)	1 (2.14)	4 (4.33)
1	2 (40)	1 (2.14)	3 (25)
2	0 (0)	2 (7.28)	2 (6.16)
3	0 (0)	3 (9.42)	3 (25)
Total (for each variable)	5 (100)	7 (100)	12 (100)

**Table 2:** Demographic characteristics of key informants of study

Variable	N	%
Age		
Under 40 years old	4	5.44
40 years old and older	5	5.55
Level of education		
bachelor	5	5.55
master	3	4.33
PhD	1	1.11
Job		
Midwife of maternity ward	3	3.33
Midwife of health base	1	1.11
Midwife of the Maternal Health Department	1	1.11
University faculty member	2	3.22
Clergyman	2	1.11
Employment history		
< 6 years	1	1.11
6–10 years	3	3.33
11–15 years	2	3.22
Over 15 years	3	3.33
Total (for each variable)	9	100

**Table 3:** Final codes, sub- categories and main categories extracted from the study

Main categories	Sub-categories	Code
Incentives	Individual factors	Enthusiasm for having children
		Desirable level of knowledge of awareness
		Individual responsibility
		Positive attitude
	Family factors	Accompanying families
		Optimal interaction between couples
	Economic factors	Sustainable financing
		Free childbearing services
	Legislative factors	Implementing a health transformation plan
		The supportive role of officials and legislators
	Organizational factors	Factors related to health care providers
		Physical structure appropriate to health care recipients
Constraints	Individual factors	Emotional-social immaturity
		Lack of awareness and knowledge
		High-risk spouse behaviors
		Misunderstanding between couples
	Organizational factors	Human resource constraints
		Allocated budget constraints
		Improper physical structure
	Socio-economic factors	Economic insecurity
		Lifestyle changes compared to past
		Changing roles
	Legislative factors	Defect in existing rules
		Lack of supportive rules
		Lack of integrated enforcement of relevant rules
Authoritarian gender attitudes	Subjective norms	Other important people
		Media
	Stereotypes	Social conformity
		Habits
		Sociability
	Hidden fears	Fear of judgment
		Fear being rejected
		Fear of power inverse

### Incentives

Besides some of the barriers that reduce men's involvement in prenatal, childbirth, and postpartum cares, a number of individual, family, economic, organizational, and legislative factors increase men’s involvement. This class consists of 5 sub-categories, including: individual incentives, family incentives, economic incentives, legislative incentives, and organizational incentives.

### Individual factors

The passion and interest of some men to become father was a factor that was considered by the participants in this study as a facilitator of men’s involvement. Some of them believed that some men have good involvement because they are well aware of the importance of their involvement and issues related to prenatal and childbirth and postpartum cares. Individual responsibility and Positive attitude were other Individual factors that were stated by the participants.

“He is helping his wife for nine months. He is helping his wife for his desire to have a child” (Participant No. 19, self-employed, Diploma, 60 years old).

### Family factors

Based on the participants, men who are encouraged to involve by their primary family (father, mother, siblings) have better involvement and cooperation with their spouses in midwifery care. Some participants in this study believed that proper relationships between couples and the expression of desires by women increase the men’s involvement.

“My mother-in-law also tells him be careful of your wife.” (Participant No. 8, Pregnant Woman, bachelor, 35 years old).

### Economic factors

Most participants believed that despite the high cost of living and the high cost of midwifery services, if a man does not have a reliable and sustainable financial source, he would not have an opportunity and motivation to help his wife. In contrast, having a good income and a reliable and stable financial source will be an incentive for his involvement. Some participants were satisfied with the free provision of some services during pregnancy and childbirth and after childbirth and thus increased men’s involvement, and hoped that such services to increase, especially for low-income families in the community.

“My brother helps his wife so much and it is due to his income. He has high income. He helps his wife and children in spending." (Participant No. 19, male, self-employed, diploma, 60 years old).

### Legislative factors

Some participants in this study believed that health transformation plan and reduction of midwifery service costs as an important turning point in increasing men’s involvement in midwifery cares. The cooperation and support of some managers of centers and political officials in increasing men’s involvement was one of the facilitating factors mentioned by some key informants.

“Fortunately, health transformation plan has reduced the costs significantly, including costs of maternity, hospitalization, and tests. We are currently performing some of our tests in comprehensive health centers for free” (Participant No. 4, female, Head of Maternal Health department, master, 44 years old).

### Organizational factors

Some participants believed that adequate skills of midwifery providers were influential factors in increasing men’s involvement. The appropriate space of some centers, good facilities of the centers and the separation of the rooms were among the factors that were mentioned as facilitators of men’s involvement by key informants of this study.

"our other bases are very good, for example, Safashahr center, all are good rooms. For example, In Meysam center, all rooms are partitioned" (participant No. 16, midwife of health base, bachelor, 33 years old).

### Constraints

According to participants, in addition to authoritarian gender attitudes, a number of individual, economic, organizational, and legislative factors can also reduce men’s involvement in prenatal, childbirth, and postpartum care. This class consists of 4 sub- categories, including individual constraints, organizational constraints, socio-economic constraints, and legislative constraints.

### Individual factors

Some participants stated that problems in emotional and social personality of some men could be a barrier to their involvement in midwifery care. According to the majority of participants in this study, men's non- involvement was due to lack of knowledge about issues related to pregnancy and childbirth and changes and needs of women in this period and lack of familiarity with way of participating and so on. The presence of high-risk behaviors such as addiction, leaving life, remarriage, inappropriate behavior of women, etc. are among the factors that were mentioned as reducing factors in men’s involvement by some participants.

"They don't know that a woman has these needs, and in this way, for example, they can meet their wife's needs." (Participant No. 5, female, faculty member, PhD, 41 years old).

According to some participants, marital conflicts and misunderstandings between couples can negatively affect men’s involvement.

"I think their cultures must be adapted with each other. I always say it might take three to five years so that couples culture to be adapted with each other. If a woman has a sensitive spirit, when her husband's family says something might be important for her, while it does not important for man. They need to be adapted with each other "(Participant No. 13, midwife and instructor of childbirth preparation classes, bachelor, 41 years old).

### Organizational factors

The low number of midwifery service providers in each work shift, despite the large volume of works, was one of the barriers to men’s involvement, which was mentioned by some key informants of this study. Some of the key informants participating in this study were complaint of the budget allocated to centers and considered it a barrier to men's financial involvement in midwifery care.

"Of course, we have a budget constraint in this regard and we have a cost ceiling to introduce low-income people" (Participant No. 4, female, head of the Maternal Health Department, Master, 44 years old).

Small space, lack of separating rooms and low number of seats in health service centers are among the organizational factors that were mentioned as barriers of men’s involvement by some key informants of this study.

### Socio-economic factors

According to the participants, lack of job security in men and the possibility of dismissal from work, if they take leave to accompany their spouse, as well as high cost of services during this period are among the factors making men prefer their job over accompanying their spouse. Some participants believed that changing the lifestyle of families compared to the past, high costs and concerns of today's lives have caused men to have less opportunities and motivation to help their spouse.

"In old days, only men managed it well. They did not have today's concerns (Participant No. 19, male, self-employed, diploma, 60 years old).

Some participants considered change in the roles and responsibilities of men and women in today's community and increasing men's expectations of women, even to fulfill men's responsibilities, as a barrier to men’s involvement.

"Unfortunately, both before and after childbirth, the only expectation is from the spouse, while the duty and role of the man is forgotten" (Participant No. 9, clergyman, 35 years old).

### Legislative factors

Some participants believed that the rules to protect men and increase their involvement are defected and need to be reformed. Some participants stated that there were no rules to support men’s involvement, and even the rules that had previously been passed in this regard were removed after a while. Also, some participants complained of lack of coordinated and integrated implementation of some rules between different public and private centers.

"The two-week postpartum leave rule considered for men was very good and helpful in that regard, but unfortunately it was removed." (Participant No. 4, female, Head of Maternal Health Department, Master, 44 years old).

"In private hospitals, the presence of men is not a problem, but in public hospitals, conditions are different" (Participant No. 9, clergyman, master, 35 years old).

### Gender authoritarian attitude

Considering male gender as a superior gender is one of the factors that are effective in men’s involvement by most key informants. This class consists of 3 sub- categories, including subjective norms, stereotypes, and hidden fears.

### Subjective norms

The subjective norm refers to the social pressure perceived by the individual to do or not to do the desired behavior. Individuals do often according to their perception of others (friends, family, co-workers, etc.) [18]. Models that men adopt them as norm are extremely influential in their participatory behaviors. These norms can be family, friends, acquaintances, or the media, whether real or virtual media. Some participants referred to high effect of man’s primary family (mother, father, and siblings), friends and relatives on men’s participatory behavior and TV series and movies in creating authoritative attitudes in men and reducing their involvement.

"I know someone who has a PhD level of education but when I said these things in class, his mother said, 'What do you expect men to do? Don't say that to men. It is clear that the family is reminding the man not to help his wife." (Participant No. 13, midwife of the maternity ward and instructor of preparation classes for childbirth, bachelor, 41 years old).

### Stereotypes

People stereotypes and beliefs about men have a great effect on men’s involvement. Some participants believed that some men, as a result of following the values ​​and behaviors of the majority of society, agreed with them and considered it shameful to involve and accompany their spouse. Also, some of the habits of men reduce their involvement in prenatal, childbirth and postpartum care.

"In private conversations with each other, working at home and taking care of their child is a shame and disgrace" (Participant No. 5, female, faculty member, PhD, 41 years old).

### Hidden fears

According to some participants, some men refuse to involve because of the possibility of negative judgments about them, fear of rejection by those around them and fear of power inversion (they have to do their wife’s duties and responsibilities).

"Someone may like to work, to collaborate, but he says to himself, 'I'm a man, it is non-accepted to do that work (Participant No. 11, midwife of the maternity ward, bachelor, 40 years old).

## Discussion

The aim of this qualitative study was to explain the barriers and facilitators of Iranian men’s involvement in perinatal care. The results of this study are presented in three main categories of incentives, constraints, and authoritarian gender attitudes.

Review of the subjects expressions and statements in the class of incentives shows that from the perspective of pregnant women or women who have given birth recently, men and key informants of this study, along with factors such as desire and enthusiasm to be father and having a positive attitude towards it, support of families and desirable interaction between husband and wife, having a sustainable source of financial support and the supportive role of officials and service providers, and proper structure of health centers have a significant impact on promoting men’s involvement during pregnancy, childbirth and postpartum periods. The results of studies conducted by Mortazavi, Mirzaei, Aborigo et al. support those of our study [11]. Having an appropriate model and defining a clear role for men were among the factors influencing their parental role. The support of other people, such as spouses, family, and friends, and the formation of male support groups can play a major role in this regard [20], which is unfortunately often overlooked [19]. Also, work environments can be a good source of support for fathers who, in addition to providing information support and raising men's awareness, help men to play a paternal role by providing financial support and creating flexible working hours [20].

Another main class of this study was the constraints that reduced men’s involvement. Lack of knowledge and awareness, lack of emotional-social maturity and high-risk behaviors of men, conflicts between husband and wife, lack of sense of economic security in men, change of roles and lifestyles compared to the past, limited budget and human resources, improper physical structure of health centers, unwillingness and threatening behavior of health care providers and defects in social rules were among the barriers to men’s involvement during the perinatal period based on expression of participants in the present study. These results are consistent with the results of many similar studies [12, 21–25].

Participants of the present study, like some studies, reported job issues and economic problems as one of the most important barriers to men’s involvement. Prolonged and time-consuming administrative procedures for providing perinatal services to women prevent their husbands to wait for their wives for long periods of time for fear of losing their jobs or low wages. Despite their inner desire, they are unable to accompany their spouse to receive health care. The results of the study conducted by Nesane et al. confirm this issue [22].

Statements made by the participants in the class of authoritarian gender attitudes in the present study indicated that subjective norms of people around them and the media, the stereotypes of society, and men’s hidden fears of being judged, rejected, and inversed power in family were the factors that could influence authoritarian gender attitudes in society. Pregnancy and childbirth were also considered to be one of the gender processes that involvement in issues and problems related to it is unique to women [26]. In addition to the promising results of some studies on changing norms of societies and changing men's behaviors over time and increasing their presence in prenatal or postpartum cares, negative attitudes governing the society in many cultures still prevent active men’s involvement, as this issue has been confirmed in other studies [27]. Although reforming the negative norms governing societies and institutionalizing and promoting a culture of men’s involvement in perinatal care is obvious and necessary, it will certainly not be possible without public education and the support of community leaders and NGOs [24].

This study, despite the emergence of new results, also suffer some limitations. For example, some men’s non-willingness to participate in the study was one of the limitations of the study, although the researcher tried to solve this problem by explaining the objectives of the study to them and to ensure confidentiality of information and interviews at their desirable time and place. In addition, non-willingness of some of them to talk too much and willingness to give short answers was another limitation of the study, which researcher tried to get more detailed answers by asking more open-ended questions.

**Fig. 1:**
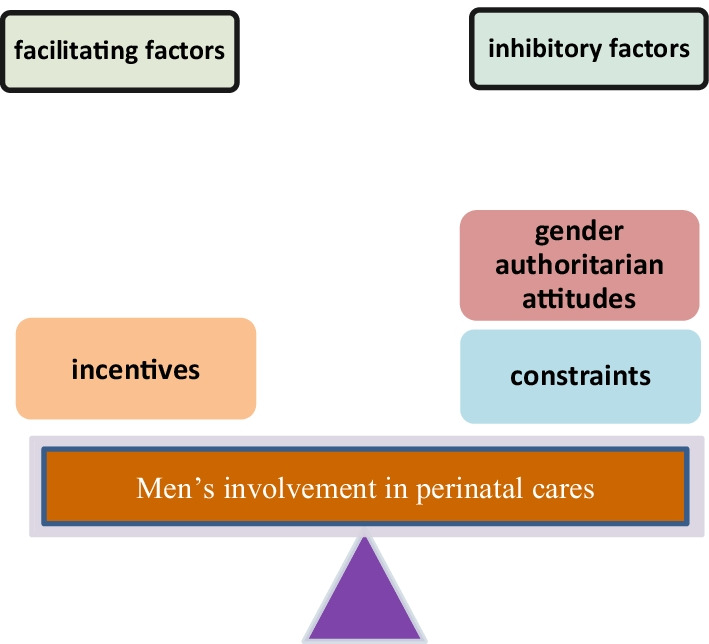
Conceptual model of factors affecting men’s involvement in perinatal care

In general, the present study was conducted to determine the barriers and facilitators of Iranian men's involvement in perinatal care. Based on the findings of this study, a set of dual mechanisms can effect on men’s involvement in perinatal care, in which authoritarian gender attitudes and individual, organizational, socio-economic and legislative constraints prevent men’s involvement in these cares, while individual, family, economic, and organizational incentives facilitate such behaviors (Fig. 1). The results revealed that many men, despite their inner desire, can not involve properly with their spouse in the perinatal period due to barriers such as social norms, lack of knowledge and awareness, economic pressures, problems in health care centers and lack of support from officials and policymakers, etc.. It is recommended health officials to pay attention to designing appropriate training programs for men and perinatal care providers, planning to promote men’s involvement culture to enhance maternal health, designing and implementing supportive policies and efforts to remove existing barriers.

## Conclusions

The present study was conducted to determine the barriers and facilitators of Iranian men's involvement in perinatal care. Based on the findings of this study, men face a dual mechanism in participating in perinatal care, in which some of these factors (incentives such as having a sustainable source of financial support, the supportive role of providers, proper structure of health centers, etc.) can facilitate their participation and others (constraints such as Lack of awareness, lack of emotional-social maturity, etc., and authoritarian gender attitudes such as the stereotypes of society, men’s hidden fears of being judged, inversed power in family, etc.) can reduce it.

## Data Availability

The datasets used and/or analyzed during the current study are available from the corresponding author on reasonable request.

## Abbreviation

ICPD: International Conference on Population and Development
